# Luminescence lifetime thermometers based on hybrid cuprous halides with exceptional water resistance and giant thermal expansion

**DOI:** 10.1038/s41377-025-01910-1

**Published:** 2025-06-24

**Authors:** Chenliang Li, Luping Wang, Datao Tu, Xiaoying Shang, Mingjie Yang, Jiacheng Gong, Fei Wen, Yun Xing, Zhi Xie, Jiaxin Jiang, Shaohua Yu, Xueyuan Chen

**Affiliations:** 1 State Key Laboratory of Structural Chemistry, and Fujian Key Laboratory of Nanomaterials, Fujian Institute of Research on the Structure of Matter, Chinese Academy of Sciences, Fuzhou, Fujian 350002 China; 2 Fujian Science & Technology Innovation Laboratory for Optoelectronic Information of China, Fuzhou, Fujian 350108 China; 3 School of Physical Science and Technology, ShanghaiTech University, Shanghai, 201210 China; 4 Fujian College, University of Chinese Academy of Sciences, Fuzhou, Fujian 350002 China; 5 Strait Institute of Flexible Electronics (SIFE, Future Technologies), Fujian Key Laboratory of Flexible Electronics, Fujian Normal University and Strait Laboratory of Flexible Electronics (SLoFE), Fuzhou, 350117 China; 6 College of Mechanical and Electronic Engineering, Fujian Agriculture and Forestry University, Fuzhou, Fujian 350100 China

**Keywords:** Optical sensors, Imaging and sensing

## Abstract

Optical probes hold great promise for temperature sensing owing to their attractive properties including rapid response, high spatial resolution, and remote non-invasive detection. However, the exploration of thermometric probes is hindered by their low relative sensitivity (*S*_*r*_) or poor structural stability in water. Herein, we propose the first example of organic-inorganic metal halides based on TPP_3_Cu_2_Br_2_ (TPP = triphenylphosphine) that simultaneously present excellent water resistance and sensitive temperature-dependent photoluminescence lifetime in water. Benefiting from the soft lattice induced by the organic molecule of TPP, giant thermal expansion and great lattice distortion were achieved with increasing temperature. As such, the self-trapped exciton luminescence lifetime of TPP_3_Cu_2_Br_2_ can be shortened to 1.9% of the initial value from 280 to 380 K, resulting in the highest *S*_*r*_ of 12.82% K^−1^ among the undoped metal halides based luminescent thermometers. Significantly, TPP_3_Cu_2_Br_2_ displayed extraordinary water stability with emission intensity remaining nearly unchanged after immersing in water for 15 days. Moreover, high-precision luminescence lifetime based thermal sensing in water environment was successfully conducted, which proved to be inert to the detection depth in water with a small read-out error. This work offers new routes in the exploration of novel metal halides for highly sensitive thermometric probes toward versatile application scenarios.

## Abstract (web summary)

TPP endows TPP_3_Cu_2_Br_2_ with both excellent water resistance and sensitive temperature-dependent luminescence lifetime, achieving a *S*_*r*_ up to 12.82% K^−1^.

## Graphical abstract

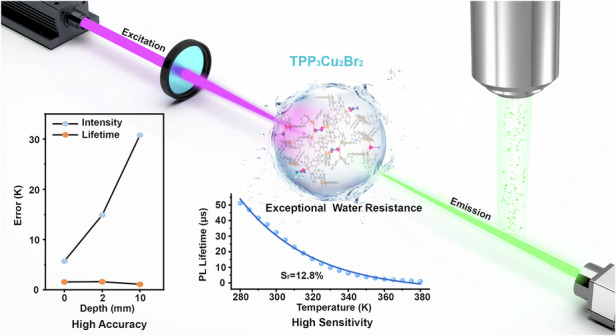

## Introduction

Optical thermometer is widely employed for temperature sensing by monitoring optical parameters like temperature-dependent changes in photoluminescence (PL) intensity^1–7^ or PL lifetime^8–10^. Nevertheless, intensity-based optical thermometers like infrared thermal imager are susceptible to various factors including light source, sample concentration, photobleaching, scattering, and absorption at different wavelengths^11–14^. PL lifetime proves advantageous for precise temperature sensing as it remains unaffected by the aforementioned factors. As such, PL lifetime-based thermometry fundamentally circumvents the limitations inherent to the conventional infrared thermal imager^15^. Currently, temperature sensing based on PL lifetime mainly employs Ln^3+^ ions and ns^2^ ions doped phosphors. For Ln^3+^-doped phosphors, the 4*f*-4*f* transitions of Ln^3+^ ions are not sensitive to surroundings due to the shielding of 5*s*5*p* shell, therefore exhibiting a small PL lifetime variation with temperature and a low temperature sensing sensitivity^16^. Although ns^2^ ions doped phosphors tend to display higher temperature sensing sensitivity, their lifetimes are generally located in the ns range^17–20^. Such a small PL lifetime requires sophisticated instrumentation and short-pulsed lasers, which is not feasible for most practical scenarios. Therefore, there is an urgent need to develop novel materials with large PL lifetime variation ability for sensitive temperature sensing.

In the past decade, metal halides (MHs) have received increasing attention for PL lifetime-based temperature sensing^21–26^. Particularly, some organic-inorganic metal halides (OIMHs) have significant temperature-dependent structural properties. At high temperatures, the thermal movement of organic molecules causes dramatic expansion of the crystal cells. Such crystal expansion induces the formation of defects, leading to a temperature-dependent nature of the PL lifetime. These OIMHs can provide sensitive temperature sensing based on self-trapped exciton (STE) luminescence with microseconds or longer PL lifetime, making them very suitable for applications in PL lifetime-based thermometry^27–30^. However, their instability in water severely limits their application in humid environments^31–33^. Despite substantial advancements being made in OIMHs, it remains challenging to explore OIMHs that are water-stable and highly temperature-sensitive in PL lifetime.

One possible strategy to address this challenge is introducing hydrophobic organic molecules into the OIMHs. Herein, we propose a novel kind of OIMH based on TPP_3_Cu_2_Br_2_ (TPP = Triphenylphosphonium), presenting exceptional water stability as sensitive luminescence lifetime thermometers in water environment. Upon excitation with ultraviolet (UV) light, TPP_3_Cu_2_Br_2_ emitted bright green STE luminescence with a PL quantum yield (PLQY) of 41.5%. Benefitting from its excellent temperature-dependent PL lifetime, a maximal relative sensitivity (*S*_*r*_) of 12.82% K^−1^ at 380 K for temperature sensing was achieved. Moreover, TPP_3_Cu_2_Br_2_ exhibited excellent water stability, whose PL intensity remained 97.3% of the initial value after 15 days of soaking in the water. We revealed that such excellent luminescent stability was owing to the protective effect of the hydrophobic organic molecules. Furthermore, we demonstrated the application of TPP_3_Cu_2_Br_2_ for high-precision thermal sensing in the water environment.

## Results

### Crystal Structure

**Fig. 1:**
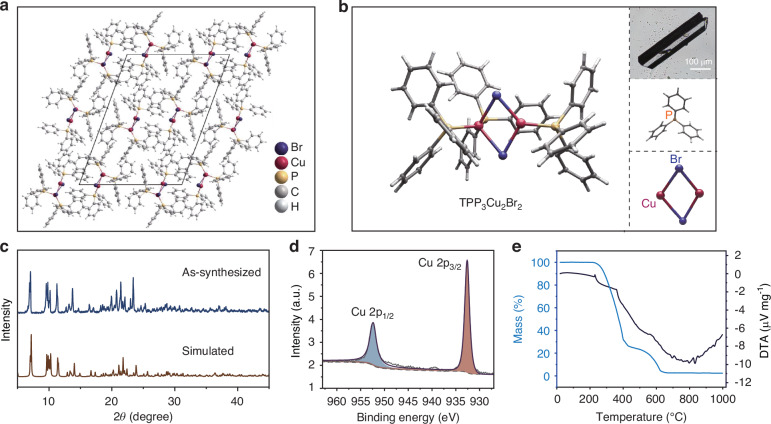
Structural characteristic of TPP_3_Cu_2_Br_2_. **a** Single crystal structure of TPP_3_Cu_2_Br_2_. **b** Schematic diagram of the structure of TPP_3_Cu_2_Br_2_. Inset: Micrograph of the TPP_3_Cu_2_Br_2_ single crystal under ambient light, schematic diagram of the structure of triphenylphosphine and [Cu_2_Br_2_] dimer. **c** PXRD pattern of TPP_3_Cu_2_Br_2_ and simulated pattern from the single crystal. **d** High-resolution XPS spectrum of Cu 2p in TPP_3_Cu_2_Br_2_. **e** TGA and DTA curves for the TPP_3_Cu_2_Br_2_ powder

TPP_3_Cu_2_Br_2_ was synthesized by a simple solvothermal method from the precursors of TPP-HBr and CuBr in a mixed solvent of N,N-Dimethylformamide and H_3_PO_2_, resulting in subcentimeter transparent acicular crystals with green emission upon 365 nm excitation (Fig. S1, Supporting Information). The crystal structure of TPP_3_Cu_2_Br_2_ was obtained by resolving single-crystal X-ray diffraction (SCXRD) data (Fig. S2 and Tables S1–S4, Supporting Information). It was determined that TPP_3_Cu_2_Br_2_ crystallized in a monoclinic phase with P2_1_/n space group, where a = 19.0348(2) Å, b = 9.87200(10) Å and c = 26.1822(3) Å. The [Cu_2_Br_2_] dimer is covalently bonded to the P atom in the TPP molecule via the Cu atom, which isolates the [Cu_2_Br_2_] dimer and constitutes the 0D structure (Fig. 1a, b). The bond lengths of Cu-P and Cu-Br are distributed in the range of 2.193–2.563 Å, whereas the Cu-Cu distance in the nearest dimer is 8.370 Å. Such a large distance also elucidated the isolated 0D structure of TPP_3_Cu_2_Br_2_. Powder X-ray diffraction (PXRD) pattern of the as-synthesized crystals agreed well with the simulated structure from single crystal data (Fig. 1c). Meanwhile, X-ray photoelectron spectroscopy (XPS) was used to probe the chemical nature of TPP_3_Cu_2_Br_2_. The characteristic peaks of C, P, Cu, and Br were identified, and the peaks of Cu 2*p*_1/2_ (952.4 eV) and 2*p*_3/2_ (932.6 eV) confirmed the monovalent state of Cu (Fig. 1d and Fig. S3, Supporting Information). Scanning electron microscopy (SEM) showed that the crystals displayed an acicular shape. Energy dispersive X-ray (EDX) spectrum mapping confirmed a uniform distribution of the elements Cu, P, and Br (Fig. S4, Supporting Information). In addition, the characteristic peaks located at 3053 and 1434 cm^−1^ in the Fourier transform infrared (FTIR) spectrum were attributed to C-H stretching vibrations and C = C vibrations of benzene, indicating that the organic groups were distributed in TPP_3_Cu_2_Br_2_ (Fig. S5, Supporting Information). Through thermogravimetric analysis (TGA) and differential thermal analysis (DTA), it was observed that the decomposition of TPP_3_Cu_2_Br_2_ started at a temperature up to 230 °C. Such good thermal stability favors their applications in high-temperature environments (Fig. 1e).

### Photoluminescence properties and mechanisms

**Fig. 2:**
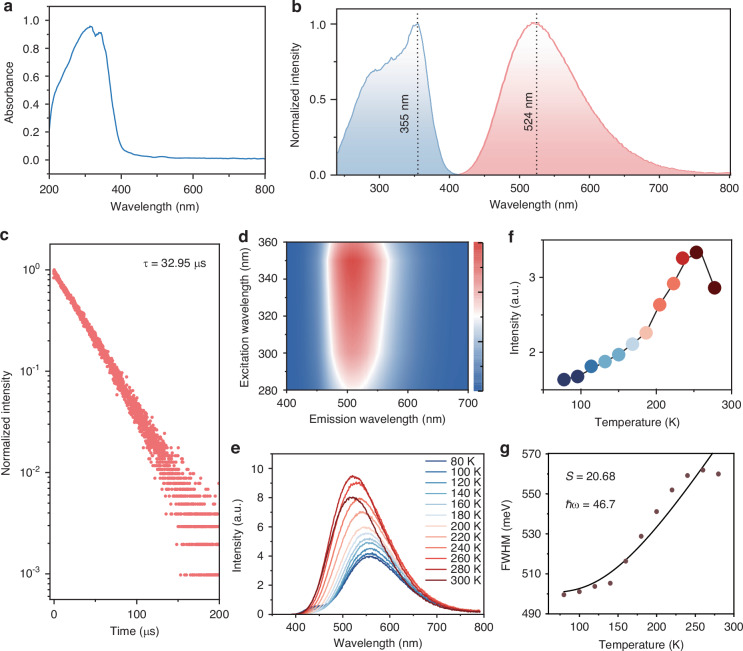
Photoluminescence characteristic of TPP_3_Cu_2_Br_2_. **a** UV-vis absorption spectrum of TPP_3_Cu_2_Br_2_. **b** Steady-state PL excitation (left) and PL emission (right) spectra of TPP_3_Cu_2_Br_2_. **c** PL decays of TPP_3_Cu_2_Br_2_ by monitoring the emission at 524 nm (λ_ex_ = 355 nm). **d** Excitation wavelength-dependent emission spectra with the excitation wavelength from 280 to 360 nm. **e** Temperature-dependent emission spectra ranging from 80 to 300 K (λ_ex_ = 355 nm). **f** Integral intensity of temperature-dependent emission spectra in the temperature range 80 to 300 K. **g** FWHM as a function of temperature. Data are fitted based on Eq. (1)

According to the absorption spectroscopy (Fig. 2a), TPP_3_Cu_2_Br_2_ displayed broad absorption across the UV region (200–400 nm) with a peak of 310 nm. The bandgap was calculated to be 3.26 eV using the Tauc plot method (Fig. S6, Supporting Information). Upon excitation at 355 nm, TPP_3_Cu_2_Br_2_ exhibited broad green emission peaking at 524 nm, with a full width at half maximum (FWHM) of 120 nm and a PLQY of 41.5% (Fig. 2b and Fig. S7, Supporting Information). The PL lifetime of TPP_3_Cu_2_Br_2_ was determined to be 32.95 μs based on mono-exponential fitting (Fig. 2c). We measured the excitation wavelength-dependent (280–360 nm) emission spectra and the emission wavelength-dependent (450–620 nm) excitation spectra, which indicated that both the normalized PL emission and excitation spectra were essentially unchanged with different wavelengths, demonstrating a single source of emission (Fig. 2d and Fig. S8, Supporting Information). Note that TPP_3_Cu_2_Br_2_ emitted strong broadband green PL while TPPBr exhibited negligible emission (Fig. S9, Supporting Information), revealing that the TPP group in TPP_3_Cu_2_Br_2_ may not act as a luminescent center.

In order to gain a deep understanding of the photophysical mechanism of TPP_3_Cu_2_Br_2_, we acquired temperature-dependent steady-state PL spectra from 80 to 300 K (Fig. 2e, f). It was discovered that the PL intensity anomalously increased with elevating the temperature from 80 to 280 K. The FWHM of the emission became progressively wider with the rising of temperature. The FWHM variation with the temperature can be fitted to derive the Huang-Ryhs factor (S) and the phonon frequency (ℏω) by using the following equation^34,35^:

1 $${\rm{FWHM}}\left(T\right)=2.36\sqrt{S}{{\hslash }}{\rm{\omega }}\sqrt{\coth \frac{{{\hslash }}{\rm{\omega }}}{2{k}_{B}T}}$$

Correspondingly, S and ℏω were determined to be 20.68 and 46.7 meV, respectively (Fig. 2g). Such a large S revealed the strong electron-phonon coupling in TPP_3_Cu_2_Br_2_^36^.

**Fig. 3:**
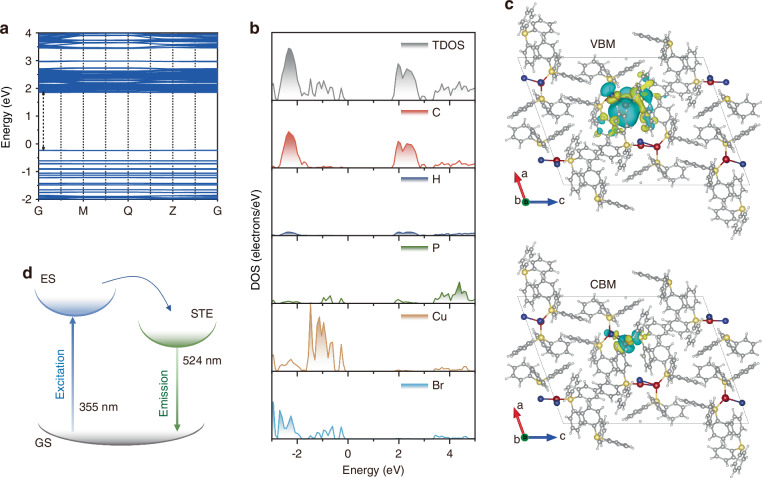
Photoluminescence mechanism of TPP_3_Cu_2_Br_2_. **a** Calculated band structure, **b** total and partial DOS of TPP_3_Cu_2_Br_2_ based on the DFT method. **c** Diagram of the partial charge density for the VBM and CBM for the excited state of TPP_3_Cu_2_Br_2_ crystals, wherein isosurfaces are electron cloud distributions. **d** Schematic mechanism of TPP_3_Cu_2_Br_2_. GS ground state; ES excited state; STE self-trapped exciton

Furthermore, we performed density functional theory (DFT) calculations to shed more light on the PL mechanism. As indicated by the projected density of states (DOS), TPP_3_Cu_2_Br_2_ has a direct bandgap (Fig. 3a). From the charge density plots of the valence band maximum (VBM) and the conduction band minimum (CBM), it can be observed that the VBM was mainly composed of the Cu 3d and Br 4p orbitals, and the CBM was composed of TPP (Fig. 3b). The majority of the electron cloud was distributed around the TPP, with a small portion distributed on [Cu_2_Br_2_] dimer (Fig. S10, Supporting Information). In addition, the charge density plots of the excited state were also modeled. Compared to the ground state, the electron cloud in the excited state was drastically concentrated towards [Cu_2_Br_2_], leading to the distortion of [Cu_2_Br_2_] dimer (Fig. 3c)^37^. Through the excited state simulation of TPP_3_Cu_2_Br_2_, theoretical lattice parameters were calculated (Table S4, Supporting Information), confirming no significant change in the lattice parameters of a and c compared to the ground state. Nevertheless, the lattice parameter b was significantly prolonged from 9.87 Å in the ground state to 9.95 Å in the excited state. As such, the lattice was distorted in the excited state, thus resulting in the emission of STE (Fig. 3d)^38–40^.

### Temperature sensing performance

**Fig. 4:**
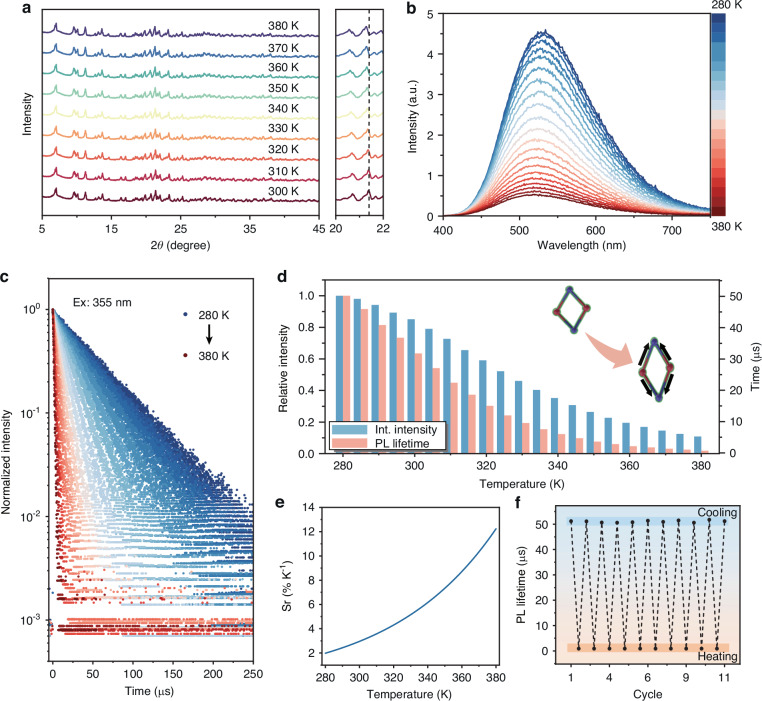
Temperature-dependent characteristic of TPP_3_Cu_2_Br_2_. **a** Temperature-dependent PXRD patterns of TPP_3_Cu_2_Br_2_ with temperature from 300 to 380 K. **b** Temperature-dependent PL spectra of TPP_3_Cu_2_Br_2_ with temperature from 280 to 380 K (λ_ex_ = 355 nm). **c** Temperature-dependent decays of TPP_3_Cu_2_Br_2_ with temperature from 280 to 380 K (λ_ex_ = 355 nm, λ_em_ = 524 nm). **d** Integrated PL intensity and PL lifetime of TPP_3_Cu_2_Br_2_ at different temperatures. **e** Calculated *S*_*r*_ values based on the PL lifetime of TPP_3_Cu_2_Br_2_ at different temperatures. f) PL lifetime of TPP_3_Cu_2_Br_2_ at 280 K and 380 K through multiple heating and cooling cycles

Organic molecular TPP endows TPP_3_Cu_2_Br_2_ with a soft lattice structure, which is sensitive to temperature change. At low temperature, the rigidity of organic molecules was strengthened, which would inhibit the deformation of [Cu_2_Br_2_] dimer and suppress the creation of the self-trapped state^41,42^. Consequently, the PL intensity of TPP_3_Cu_2_Br_2_ was substantially boosted with the temperature increased from 80 to 280 K (Fig. 2e). Meanwhile, it was found that the lattice greatly expanded with lattice volume increased by 3.6% (from 4818.3 to 4994.0 Å^3^) from 300 to 380 K (Fig. S11 and Table S5, Supporting Information), which is much higher than that (< 0.5%) of typically MHs^19,20^. The giant thermal expansion of lattice was also confirmed by the temperature-dependent PXRD of TPP_3_Cu_2_Br_2_, as evidenced by the shifting of diffraction peaks towards smaller angles with the rising of temperature (Fig. 4a). The dramatic lattice expansion with elevating temperature would introduce more defects within the crystal and thus quenching the luminescence. We compared the temperature dependence of PL intensity and lifetime from 280 to 380 K. It was discovered that the PL intensity decreased steadily as temperature increased, eventually dropping to 10.8% of its original value at 380 K (Fig. 4b). Intriguingly, the PL lifetime of TPP_3_Cu_2_Br_2_ displayed a much wider range of variation from 51.2 μs (280 K) to 0.97 μs (380 K), which was only 1.9% of the initial value (Fig. 4c, d). The PL lifetime (*τ*) and temperature (*T*) can be fitted with the following empirical exponential equation^19^:

2 $$\tau \left(T\right)=A\cdot \exp \left(\frac{T}{t}\right)+{\tau }_{0}$$

where A, t, *τ*_*0*_ are constants. Furthermore, the temperature sensing performance can be evaluated with absolute sensitivity (*S*_*a*_) and *S*_*r*_ based on the following equations:

3 $${S}_{a}=\left|\frac{{\rm{d}}\tau }{{\rm{d}}T}\right|$$

4 $${S}_{r}=\left|\frac{1}{\tau }\frac{{\rm{d}}\tau }{{\rm{d}}T}\right|$$

Accordingly, the maximum *S*_*a*_ value was determined to be 1.33 μs K^−1^ (280 K) (Fig. S12, Supporting Information), and the maximum *S*_*r*_ value was calculated to be 12.82% K^−1^ (380 K) (Fig. 4e). Note that the *S*_*r*_ value is the highest for temperature sensing among the undoped MHs. For comparison, the maximum values of *S*_*a*_ and *S*_*r*_ based on PL intensity were only 1.39% K^−1^ and 6.46% K^−1^, respectively (Figs. S13, S14, Supporting Information). Besides, the repeatability of the thermometer is a decisive parameter for its application in temperature sensing. After 11 test cycles, the PL lifetime of the TPP_3_Cu_2_Br_2_ remained essentially unchanged, confirming its good repeatability in temperature sensing (Fig. 4f). The temperature uncertainty *δT* can be calculated by using the following equation^19^:

5 $${\rm{\delta }}T=\frac{1}{{S}_{r}}\frac{{\rm{\delta }}\tau }{\tau }$$

where *δτ*/*τ* is the uncertainty of lifetime measurements. Accordingly, it was determined that TPP_3_Cu_2_Br_2_ exhibited small *δT* values (≤ 0.47 K) in the range of 280–380 K, which is beneficial for high-precision temperature sensing (Fig. S15, Supporting Information).

### Water Stability Performance

**Fig. 5:**
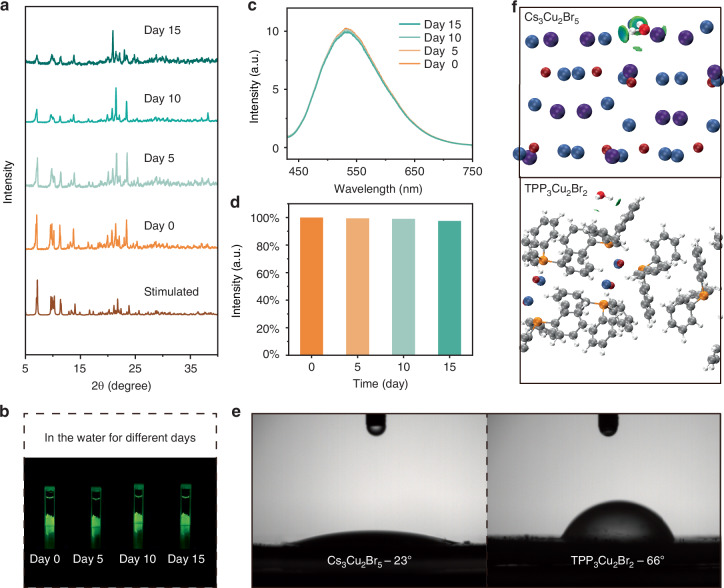
Water-stability characteristic of TPP_3_Cu_2_Br_2_. **a** PXRD patterns, **b** photographs, **c** PL emission spectra and **d** integral intensity of PL emission spectra of TPP_3_Cu_2_Br_2_ soaking in water for different days upon excitation at 365 nm. **e** Contact angle of water molecule on crystal surfaces of TPP_3_Cu_2_Br_2_ and Cs_3_Cu_2_Br_5_. **f** Theoretically calculated surface structures of H_2_O interacted with Cs_3_Cu_2_Br_5_ (up) and TPP_3_Cu_2_Br_2_ (down), wherein gray balls are C atoms, white balls are H atoms, blue balls are Cu atoms, dark red balls are Br atoms, orange balls are P atoms, purple balls are Cs atoms, red balls are O atoms. The simulation model is constructed based on water molecules on the (001) surface of TPP_3_Cu_2_Br_2_ and (040) surface of Cs_3_Cu_2_Br_5_

The PXRD pattern of TPP_3_Cu_2_Br_2_ remained in the pure phase even after immersing it in water for 15 days (Fig. 5a), indicating its excellent structural stability in water. Correspondingly, TPP_3_Cu_2_Br_2_ exhibited almost no alteration in both PL photographs and PL spectra (Fig. 5b, c). Specifically, the PL intensity was 97.3% of the initial value after 15 days, indicative of the extraordinary photostability of TPP_3_Cu_2_Br_2_ in water (Fig. 5d). To investigate the impact of TPP, we compared the surface hydrophobicity of hybrid TPP_3_Cu_2_Br_2_ and all-inorganic Cs_3_Cu_2_Br_5_. Through characterization of static-contact angle, the degree of water infiltration on TPP_3_Cu_2_Br_2_ and Cs_3_Cu_2_Br_5_ was investigated (Fig. 5e). A larger contact angle of 66° was detected for TPP_3_Cu_2_Br_2_ crystals, which greatly surpassed that of 23° for the Cs_3_Cu_2_Br_5_ counterpart. Such a high contact angle indicated that the crystal surface of TPP_3_Cu_2_Br_2_ was highly hydrophobic, thus protecting the matrix from permeability and diffusion of water molecules. Through DFT calculations, the binding energy of TPP_3_Cu_2_Br_2_ and water molecules was determined to be 0.223 eV (Fig. 5f), which is much lower than that of Cs_3_Cu_2_Br_5_ and water molecules (0.882 eV), revealing weaker hydrophilicity of TPP_3_Cu_2_Br_2_ than Cs_3_Cu_2_Br_5_.

Moreover, the interaction force between atoms was reported to be proportional to their distance^43^. The rigid molecular conformation of the TPP molecule and the large steric hindrance limit the movement of water molecules, inhibiting water molecules from directly contacting with the hydrophilic [Cu_2_Br_2_] dimer in TPP_3_Cu_2_Br_2_. The closest contact distance between water molecule and TPP_3_Cu_2_Br_2_ was calculated to be 6.583 Å for the H-Br bond length, which is much longer than the H-Br distance of 2.335 Å between water molecule and Cs_3_Cu_2_Br_5_ (Fig. S16, Supporting Information). The longer contact distance between water molecule and hydrophilic [Cu_2_Br_2_] dimer endowed superior water-resistance stability. In addition, the bond type in the matrix also significantly affects the stability of the crystal^44^. The strong Cu-P covalent bond in TPP_3_Cu_2_Br_2_ results in a higher energy barrier for ionization in water, facilitating the structural stability of TPP_3_Cu_2_Br_2_. By contrast, the ionic bonds in Cs_3_Cu_2_Br_5_ are more susceptible to ionization in water, which might lead to structural disruption. Hence, the impressive resistance to water of TPP_3_Cu_2_Br_2_ renders it an ideal material as the luminescence thermometer in water environment. To the best of our knowledge, such water-resistance MHs based luminescence lifetime thermometers have never been reported before. Note that the micron-scale dimensions of TPP_3_Cu_2_Br_2_ crystals enable spatial resolution in microdomain thermal mapping, favoring diverse sensing scenarios like mapping microzone-temperature variations in water.

### Temperature sensing in water environment

**Fig. 6:**
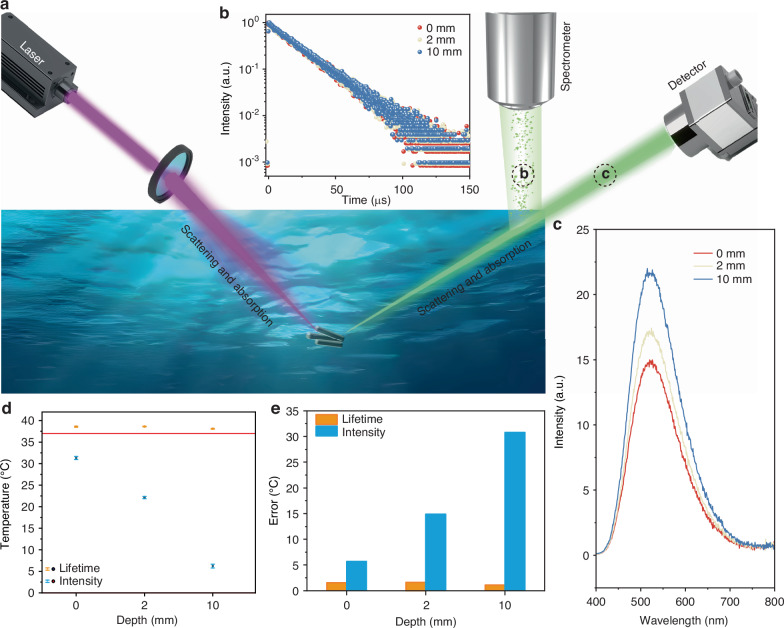
Temperature-sensing in water environment. **a** Schematic illustration of luminescence temperature-sensing based on TPP_3_Cu_2_Br_2_ soaking in water. **b** PL decays of the TPP_3_Cu_2_Br_2_ at different depths of water by monitoring the emission at 524 nm (λ_ex_ = 355 nm) at 37 °C. **c** PL emission spectra of the TPP_3_Cu_2_Br_2_ at different depths of water upon 355 nm excitation at 37 °C. **d** Comparison of calculated temperatures at different depths of water from multiple experiments based on PL intensity and PL lifetime, where data were presented as average ± standard deviation calculated from three independently measured PL emission and PL lifetime spectra. **e** Error values between calculated and actual temperatures (37 °C) at different water depths

As a proof-of-concept experiment, we applied TPP_3_Cu_2_Br_2_ for temperature sensing in aqueous solution. The PL lifetime was independently monitored three times at 37 °C (Fig. 6a). The average PL lifetime was determined to be 19.83 μs (Fig. S17, Supporting Information). Meanwhile, the PL emission spectra were monitored under the same conditions (Fig. S18, Supporting Information). The temperature can be calculated by using Eqs. 2 and 6 as standard curves, respectively.

6 $$I\left(T\right)=A\cdot \exp \left(\frac{T}{t}\right)+{I}_{0}$$

where A, t, and *I*_*0*_ are constants. Specifically, temperatures of 38.57 ± 0.13 and 31.31 ± 0.36 °C were determined on the basis of PL lifetime and PL intensity with errors of 1.57 and 5.69 °C, respectively. Therefore, PL lifetime-based temperature sensing provides higher accuracy than PL intensity-based temperature sensing.

Moreover, the absorption and scattering of light may significantly reduce the accuracy of intensity measurements at varying depths, which is unavoidable in practical applications. To this regard, we placed TPP_3_Cu_2_Br_2_ in cuvettes filled with water at varying depths to illustrate the impact of water on light interference. We monitored the PL lifetime and PL intensity of TPP_3_Cu_2_Br_2_ in different depths at 37 °C (Fig. 6b, c). The accuracy of the temperature measurements was then calculated (Fig. 6d, e). As the depth of the water increased, the PL intensity quenched significantly, while the PL lifetime remained essentially unchanged. When the water depth reached 2 mm, the error value based on PL intensity reached 14.88 °C while the value based on PL lifetime remained essentially small (1.61 °C). When the sample was immersed in 10 mm water, the detection error based on PL intensity was elevated to 30.78 °C, significantly larger than the read-out error based on PL lifetime (1.09 °C). These results highlighted that TPP_3_Cu_2_Br_2_ has great potential for accurate temperature sensing applications in water environment, offering promising opportunities for monitoring microzone-temperature variations beneath the water surface.

## Discussion

In summary, we have developed a new class of zero-dimensional hybrid cuprous halide of TPP_3_Cu_2_Br_2_, which presents excellent water resistance for sensitive temperature sensing. Specifically, TPP induced the soft lattice of TPP_3_Cu_2_Br_2_, enabling substantial lattice distortion and significant reduction of STE luminescence lifetime to 1.9% of the initial value from 280 to 380 K. Such remarkable temperature-dependent PL lifetime favored highly sensitive optical temperature sensing, exhibiting a *S*_*r*_ of 12.82% K^−1^, representing the highest value based on the undoped MHs. Furthermore, we successfully applied TPP_3_Cu_2_Br_2_ for thermal sensing with a small read-out error of 1.09 °C at a water depth of 10 mm, demonstrating the superiority of PL lifetime strategy relative to the conventional PL intensity approach. Our work reveals the great promise of TPP_3_Cu_2_Br_2_ for temperature detection in water environment, which may accelerate the exploitation of novel MHs for thermometry applications in versatile scenarios.

## Materials and methods

### Chemicals and materials

Triphenylphosphine hydrobromide (C_18_H_16_BrP, 97%) and phosphinic acid (H_3_PO_2_, 50 wt.%) were purchased from Adamas-beta Ltd. Copper(I) bromide (CuBr, 99.9%) was purchased from Aladdin (Shanghai, China). N,N-Dimethylformamide (DMF, 99.5%), and isopropanol (C_3_H_8_O, 99.7%) were purchased from Sinopharm Chemical Reagent Co (Shanghai, China). All chemicals were used without any further purification.

### Synthesis of TPP_3_Cu_2_Br_2_ single crystal

TPP_3_Cu_2_Br_2_ single crystals were prepared by a saturated crystallization method. In a typical synthesis, 1 mmol C_18_H_16_BrP and 1 mmol CuBr were dissolved in 4 mL DMF and 1 mL H_3_PO_2_ at 95 °C to form a colorless transparent solution. Then the solution was cooled to room temperature slowly. After that, the transparent crystals of TPP_3_Cu_2_Br_2_ were filtered off, washed with isopropanol to remove the solvent from the crystal surface, and dried in an oven at 60 °C. Finally, the crystals were ground into powder for further utilization.

## Supplementary information

Supplementary Information for Luminescence Lifetime Thermometers Based on Hybrid Cuprous Halides with Exceptional Water Resistance and Giant Thermal Expansion

## Data Availability

The data that support the findings of this study are available from the corresponding authors upon reasonable request.
