# Supplementary material for: Luminescence lifetime thermometers based on hybrid cuprous halides with exceptional water resistance and giant thermal expansion
Source: Light Sci Appl. 2025 Jun 24;14:224. doi: 10.1038/s41377-025-01910-1 (PMC12187932; doi:10.1038/s41377-025-01910-1)
Supplement: Supplementary file 1 — Supplementary Information for Luminescence Lifetime Thermometers Based on Hybrid Cuprous Halides with Exceptional Water Resistance and Giant Thermal Expansion [file 41377_2025_1910_MOESM1_ESM.pdf]

Light: Science & Applications

Supplementary Information for

Luminescence Lifetime Thermometers Based on Hybrid Cuprous Halides with Exceptional Water Resistance and Giant Thermal Expansion

Chenliang Li[1,2,3,4], Luping Wang[5,*], Datao Tu[1,2,4,*], Xiaoying Shang[1,2,4], Mingjie Yang[1], Jiacheng Gong[1], Fei Wen[1,4], Yun Xing[1,4], Zhi Xie[6], Jiaxin Jiang[1,4], Shaohua Yu[1,2,4] and Xueyuan Chen[1,2,3,4,*]

1 State Key Laboratory of Structural Chemistry, and Fujian Key Laboratory of Nanomaterials, Fujian Institute of Research on the Structure of Matter, Chinese Academy of Sciences, Fuzhou, Fujian 350002, China

2 Fujian Science & Technology Innovation Laboratory for Optoelectronic Information of China, Fuzhou, Fujian 350108, China

3 School of Physical Science and Technology, ShanghaiTech University, Shanghai 201210, China

4 Fujian College, University of Chinese Academy of Sciences, Fuzhou, Fujian 350002, China

5 Strait Institute of Flexible Electronics (SIFE, Future Technologies), Fujian Key Laboratory of Flexible Electronics, Fujian Normal University and Strait Laboratory of Flexible Electronics (SLoFE), Fuzhou, 350117, China

6 College of Mechanical and Electronic Engineering, Fujian Agriculture and Forestry University, Fuzhou, Fujian 350100, China

Correspondence: Luping Wang (lpwang@fjnu.edu.cn), Datao Tu (dttu@fjirsm.ac.cn), Xueyuan Chen (xchen@fjirsm.ac.cn)

Measurements

Characterization: Single-crystal X-ray diffraction (SCXRD) data were collected on a Super Nova diffractometer equipped with multilayer mirror Cu-Kα radiation (λ = 1.5418 Å) using a ω scan mode at 100 K. The structures were solved by direct methods and refined by full-matrix least-squares techniques on F^2 with SHELX-97. The crystallographic data and details of structural refinements are listed in Table S1. CCDC number 2382732 contain the supplementary crystallographic data for this paper. These data can be obtained free of charge from The Cambridge Crystallographic Data Centre via www.ccdc.cam.ac.uk/data_request/cif. Powder X-ray diffraction (PXRD) analysis was performed on an X-ray diffractometer (MiniFlex 600, Rigaku) with Cu Kα1 radiation (λ = 1.5418 Å), operating at 40 kV and 40 mA. SEM images and SEM-EDS mapping were taken with a SU8010 scanning electron microscope (SEM). X-ray photoelectron spectroscopy (XPS) measurements were performed on an ESCALAB 250Xi X-ray Photoelectron Spectroscopy using a monochromatic Al Kα source (15 kV, 20 mA). Thermogravimetric Analysis (TGA) was investigated using a NETZSCH STA449F3 simultaneous analyzer.

For optical characterization of the phosphors, the diffuse reflectance spectra were recorded on a UV–vis–NIR spectrophotometer (Lambda950, Perkin-Elmer) using $BaSO_4$ powder as the reflectance reference. The photoluminescence (PL) emission, PL excitation (PLE) spectra, and PL decays were recorded on an Edinburgh FLS980 spectrofluorometer equipped with a continuous xenon lamp (450 W), optical parametric oscillator (OPO) laser (Opolette™ HE 355 LD, OPOTEK), thermal stage for powder (77–873 K, THMS 600, Linkam Scientific Instruments) and thermal stage for liquid (280–350 K, APTC-2, Atuomatic Science Instrument). All the spectral data were recorded at RT unless otherwise noted and corrected for the response of both the spectrometer and the integrating sphere.

Computational details: The DFT calculations in this study were conducted using the Vienna Ab initio Simulation Package (VASP) code [1-4]. The ion core potentials were modeled following the projector augmented wave (PAW) approach to describe the electron−core interaction [5,6]. For electron exchange−correlation, the Perdew−Burke−Ernzerhof (PBE) functional within the generalized-gradient approximation (GGA) range was applied [7,8]. A plane wave basis set was used with a cutoff energy of 400 eV. Structural relaxations were performed until the forces on each of the atoms were below 0.02 eV $Å^{-1}$. The energies were converged to 10^{-8} eV for the electronic loop. During structural optimization and the self-consistent calculations, Gamma–Pack mesh k-space sampling grids of 2 × 4 × 2 were used to sample the Brillouin zone (BZ) [9]. To simulate the excited state of the structure, the restricted open-shell Kohn-Sham (ROKS) method implemented in the CP2K package as suggested by Wang X et al was adopted [10-15]. GTH pseudopotential and DZVP molecularly optimized basis sets were employed in the calculations [16,17]. An energy cut-off of 500 Ry was used and the delocalization error of PBE was removed by the scaled Perdew-Zunger self-interaction correction (SIC) on unpaired electrons for ROKS calculation [18-20].

Table S1. Crystal data and structure refinement for $TPP_3Cu_2Br_2$ at 100 K.

Compound	$TPP_3Cu_2Br_2$
Chemical formula	$C_{54}H_{45}Br_2Cu_2P_3$
Formula weight	1073.71
Temperature (K)	100.00(10)
λ(Å)	1.54184
Crystal system	monoclinic
Space group	$P2_1/n$
a (Å)	19.0348(2)
b (Å)	9.87200(10)
c (Å)	26.1822(3)
α (°)	90
β (°)	109.8070(10)
γ (°)	90
Volume ($Å^3$)	4628.87(9)
Z	4
ρ_{calc} (g/cm^3)	1.541
μ (mm^{-1})	4.413
F(000)	2168.0
2Θ range for data collection/°	5.022 to 151.832
Index ranges	$-23 \le h \le 23$, $-12 \le k \le 9$, $-32 \le l \le 31$
Reflections collected	33633
Independent reflections	9284 [R_{int} = 0.0204, R_{sigma} = 0.0180]
Data/restraints/parameters	9284/0/551
GOF on F^2	1.030
Final R indexes [I>=2σ (I)][a]	R1 = 0.0223, wR2 = 0.0585
Final R indexes [all data]	R1 = 0.0238, wR2 = 0.0596
Largest diff. peak/hole / e $Å^{-3}$	0.74/-0.37

[a]$R_1(F) = \sum||F_o| - |F_c||/\sum|F_o|$; $wR_2(F_o^2) = [\sum w(F_o^2 - F_c^2)^2/\sum w(F_o^2)^2]^{1/2}$

Table S2. Selected bond lengths for $TPP_3Cu_2Br_2$ at 100 K.

Atom	Atom	Length/Å
Br1	Cu3	2.5308(3)
Br1	Cu4	2.4248(3)
Br2	Cu3	2.5629(3)
Br2	Cu4	2.3652(3)
Cu3	Cu4	2.8918(3)
Cu3	P11	2.2418(4)
Cu3	P30	2.2355(4)
Cu4	P49	2.1929(4)

Table S3. Selected bond angles for $TPP_3Cu_2Br_2$ at 100 K.

Atom	Atom	Atom	Angle/°
Cu4	Br1	Cu3	71.364(9)
Cu4	Br2	Cu3	71.732(9)
Br1	Cu3	Br2	101.775(9)
Br1	Cu3	Cu4	52.611(7)
Br2	Cu3	Cu4	50.957(7)
P11	Cu3	Br1	103.250(13)
P11	Cu3	Br2	102.317(13)
P11	Cu3	Cu4	99.230(13)
P30	Cu3	Br1	112.681(14)
P30	Cu3	Br2	100.846(13)
P30	Cu3	Cu4	128.072(14)
P30	Cu3	P11	131.654(17)
Br1	Cu4	Cu3	56.025(8)
Br2	Cu4	Br1	111.198(10)
Br2	Cu4	Cu3	57.311(8)
P49	Cu4	Br1	115.437(15)
P49	Cu4	Br2	132.314(16)
P49	Cu4	Cu3	169.989(16)
C12	P11	Cu3	112.58(5)
C18	P11	Cu3	114.94(5)
C5	P11	Cu3	114.55(5)
C31	P30	Cu3	111.29(5)
C37	P30	Cu3	118.13(5)
C24	P30	Cu3	114.16(5)
C50	P49	Cu4	114.34(5)
C56	P49	Cu4	118.26(5)
C43	P49	Cu4	109.20(5)

Table S4. Crystal data for $TPP_3Cu_2Br_2$ at ground state and excited state.

	Ground state	Excited state
a (Å)	19.0348	19.03483
b (Å)	9.87200	9.94513
c (Å)	26.1822	26.18225
α (°)	90	90
β (°)	109.8070	109.8071
γ (°)	90	90
Volume ($Å^3$)	4628.87	4663.17

Table S5. Crystal data and structure refinement for $TPP_3Cu_2Br_2$ at 293 K.

Compound	$TPP_3Cu_2Br_2$
Chemical formula	$C_{54}H_{45}Br_2Cu_2P_3$
Formula weight	1073.71
Temperature (K)	293.00(2)
λ(Å)	1.54178
Crystal system	monoclinic
Space group	$P2_1/n$
a (Å)	19.4173(2)
b (Å)	9.92210(10)
c (Å)	26.6158(3)
α (°)	90
β (°)	110.0810(10)
γ (°)	90
Volume (Å^3)	4816.08(9)
Z	4
ρ_{calc} (g/cm^3)	1.481
μ (mm^{-1})	4.241
F(000)	2168.0
2Θ range for data collection/°	4.92 to 158.476
Index ranges	$-24 \leq h \leq 24$, $-9 \leq k \leq 12$, $-33 \leq l \leq 33$
Reflections collected	38100
Independent reflections	9983 [R_{int} = 0.0357, R_{sigma} = 0.0315]
Data/restraints/parameters	9983/0/551
GOF on F^2	1.064
Final R indexes [I>=2σ (I)]	R1 = 0.0293, wR2 = 0.0761
Final R indexes [all data]	R1 = 0.0339, wR2 = 0.0788
Largest diff. peak/hole / e Å^{-3}	0.41/-0.39

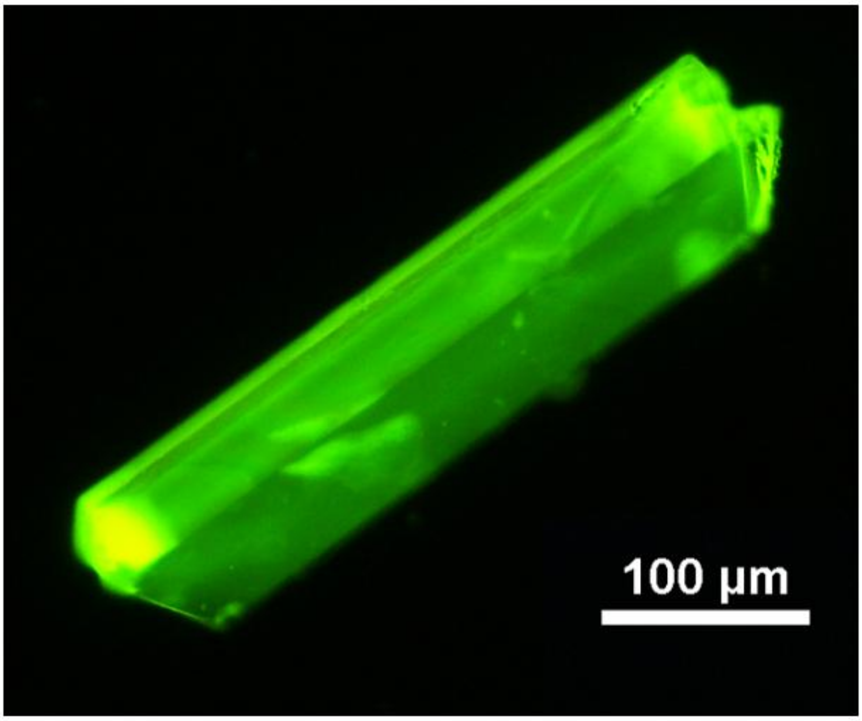

Figure S1. Micrograph of the $TPP_3Cu_2Br_2$ single crystal under 365-nm UV light.

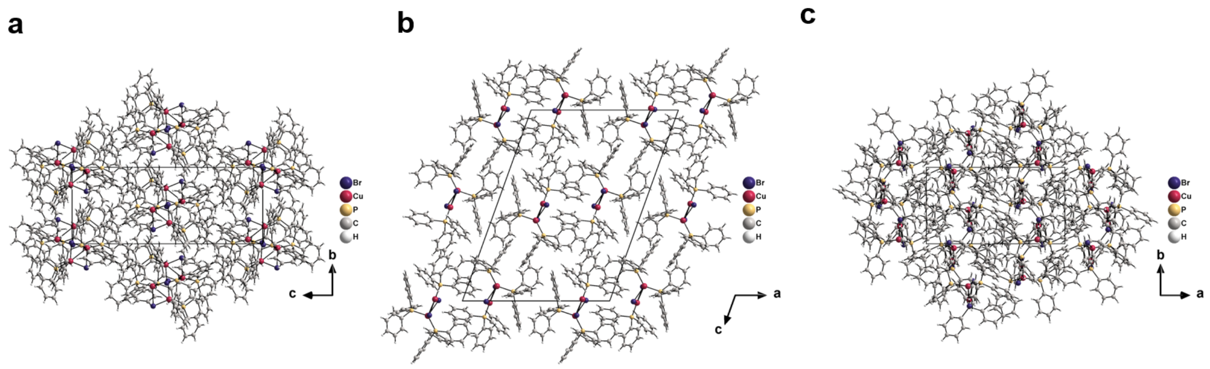

Figure S2. Crystal structure of $TPP_3Cu_2Br_2$ viewed from different angles.

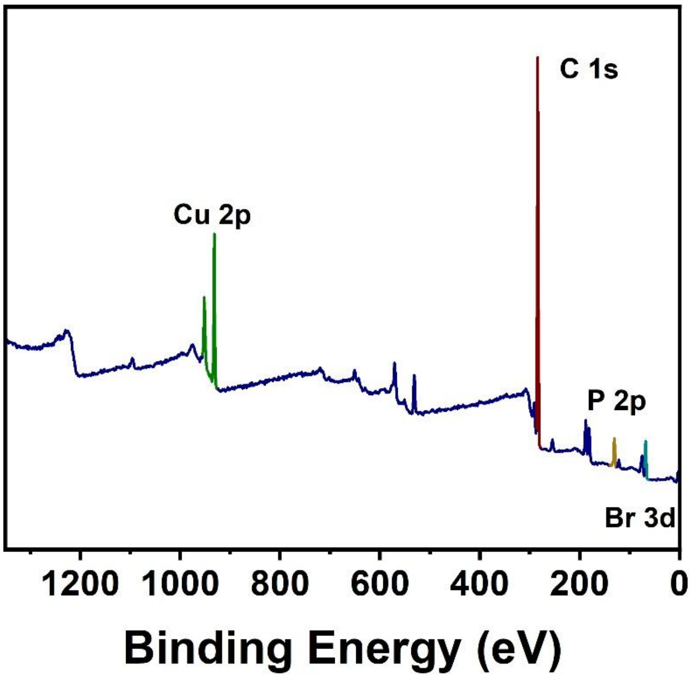

Figure S3. XPS spectrum of the $TPP_3Cu_2Br_2$ single crystal. The characteristic peaks of C, P, Cu, and Br were observed.

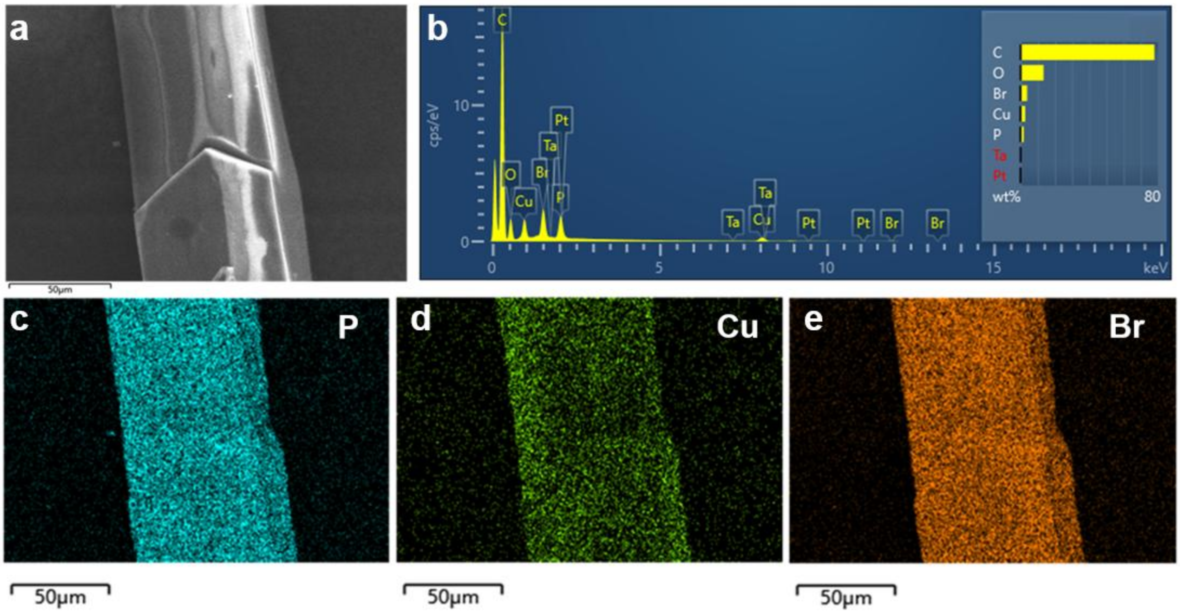

Figure S4. a) SEM images of $TPP_3Cu_2Br_2$. b) EDS spectrum of the selected point. EDS elemental mappings of c) P, d) Cu and e) Br elements in a $TPP_3Cu_2Br_2$.

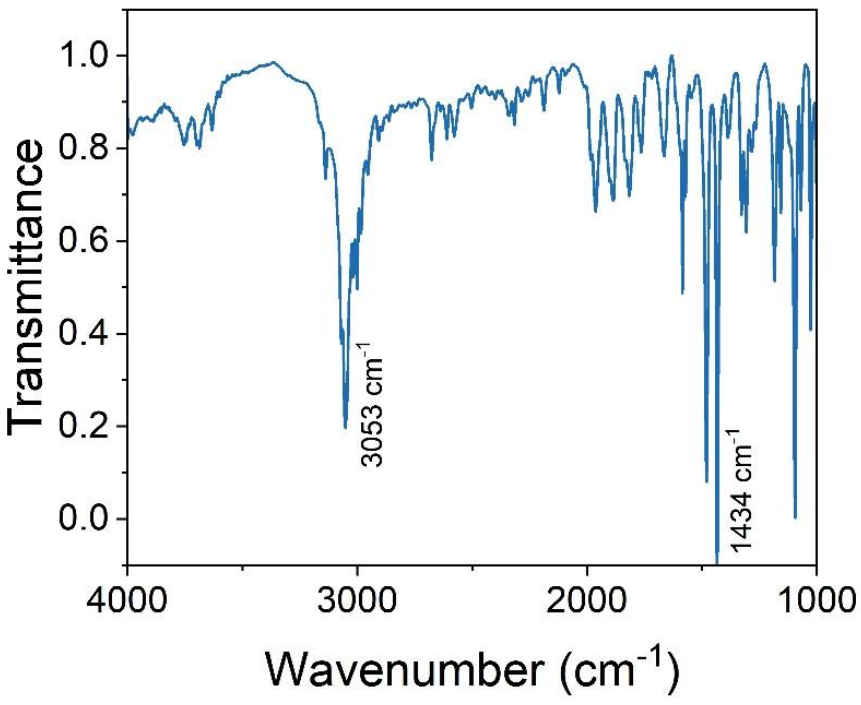

Figure S5. FTIR spectrum of $TPP_3Cu_2Br_2$. The characteristic peaks located at 3053 and 1434 cm^{-1} in the FTIR spectrum were attributed to C-H stretching vibrations and C=C vibrations of benzene, respectively.

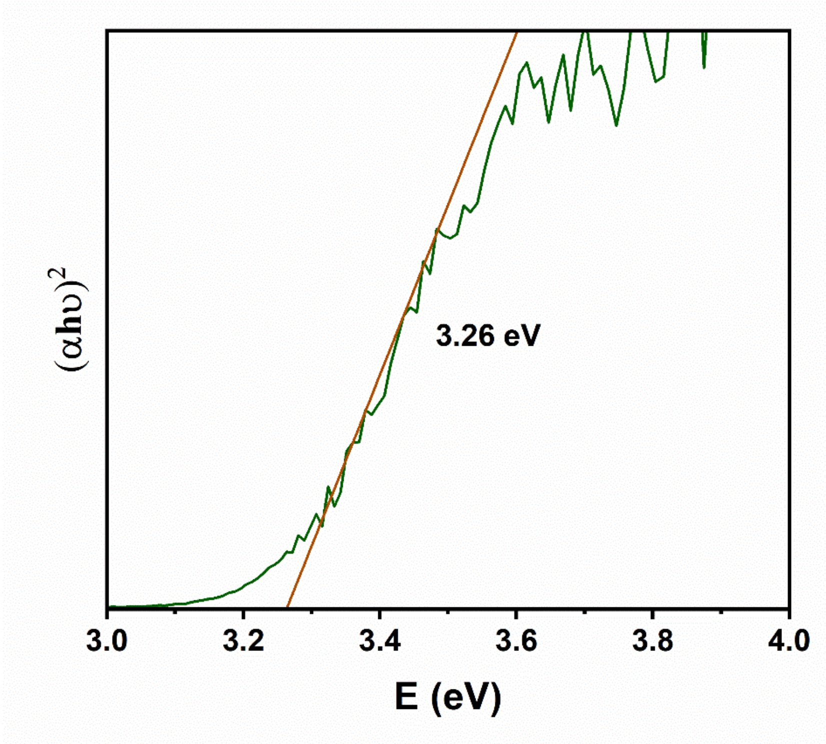

Figure S6. Tauc plot of $TPP_3Cu_2Br_2$ according to the UV−vis absorption spectra. The band gap was calculated to be 3.26 eV using the Tauc plot method.

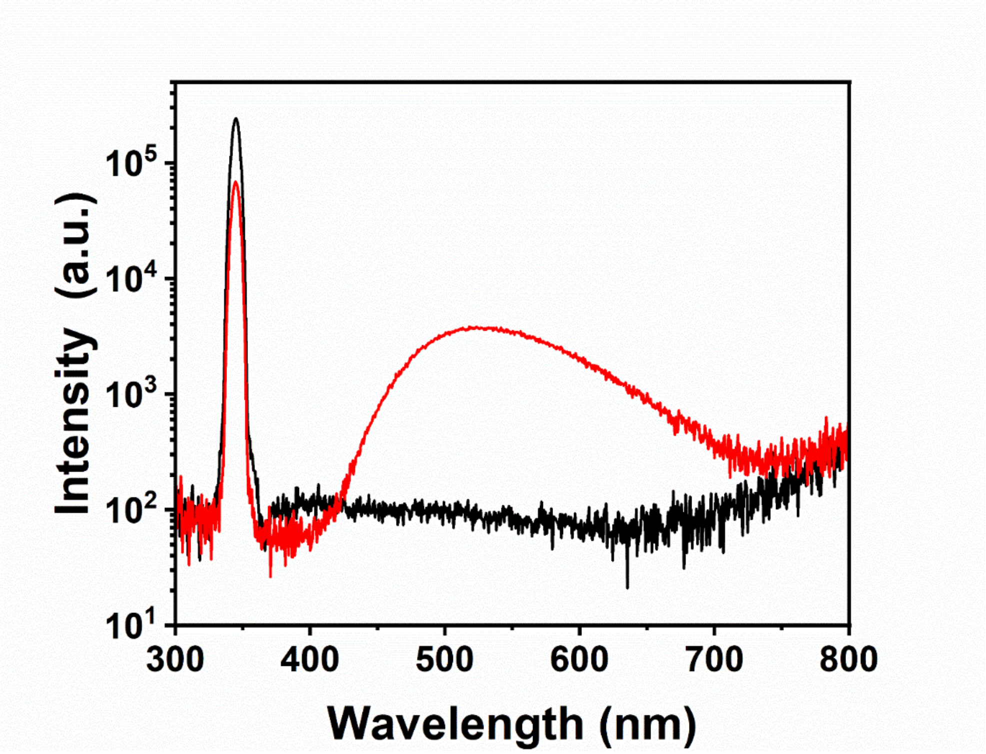

Figure S7. Absolute photoluminescence quantum yield of $TPP_3Cu_2Br_2$. Upon excitation at 355 nm, the PLQY of $TPP_3Cu_2Br_2$ was determined to be 41.5%.

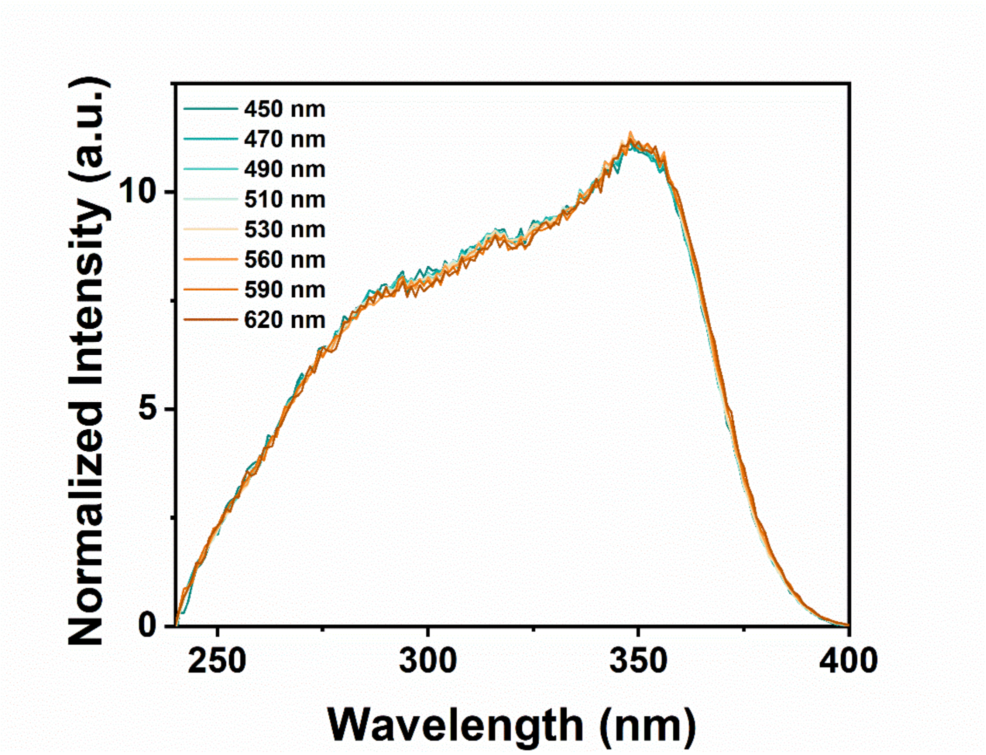

Figure S8. Emission wavelength-dependent excitation spectra with the emission wavelength from 450 to 620 nm. It was discovered that both the peak and the shape of the PLE spectra were essentially unchanged with different wavelengths.

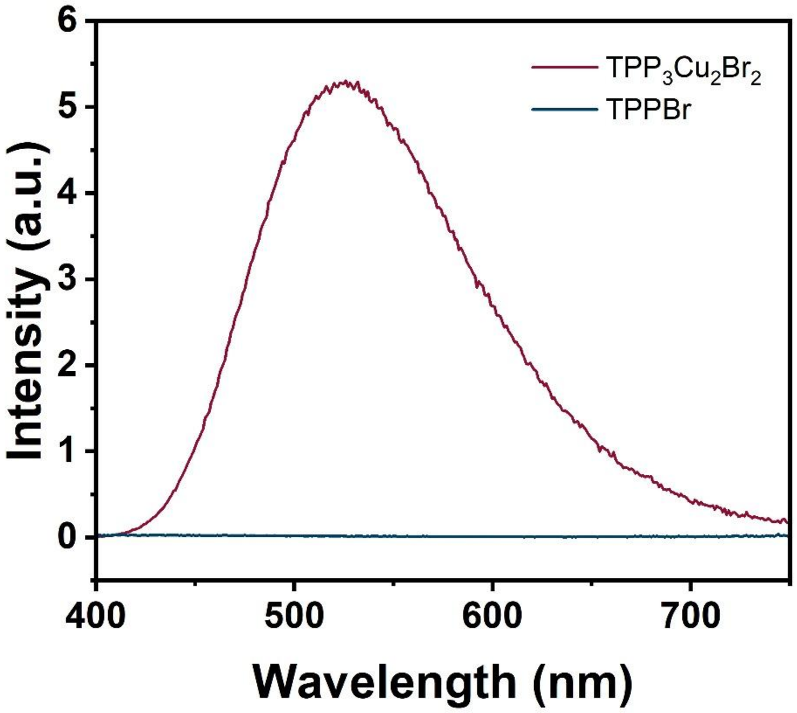

Figure S9. PL emission spectra of $TPP_3Cu_2Br_2$ and TPPBr upon excitation at 355 nm. Under identical excitation conditions, $TPP_3Cu_2Br_2$ emitted strong broadband green PL, while TPPBr exhibited negligible emission.

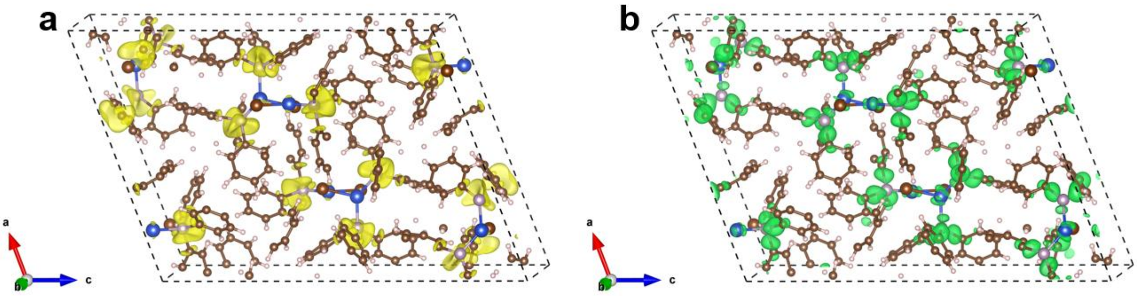

Figure S10. Diagram of the partial charge density for the a) VBM and b) CBM for the $TPP_3Cu_2Br_2$ crystal. Yellow and green isosurfaces are electron cloud distributions, respectively. The VBM was composed of the Cu 3d and Br 4p orbitals, and the CBM was composed of TPP.

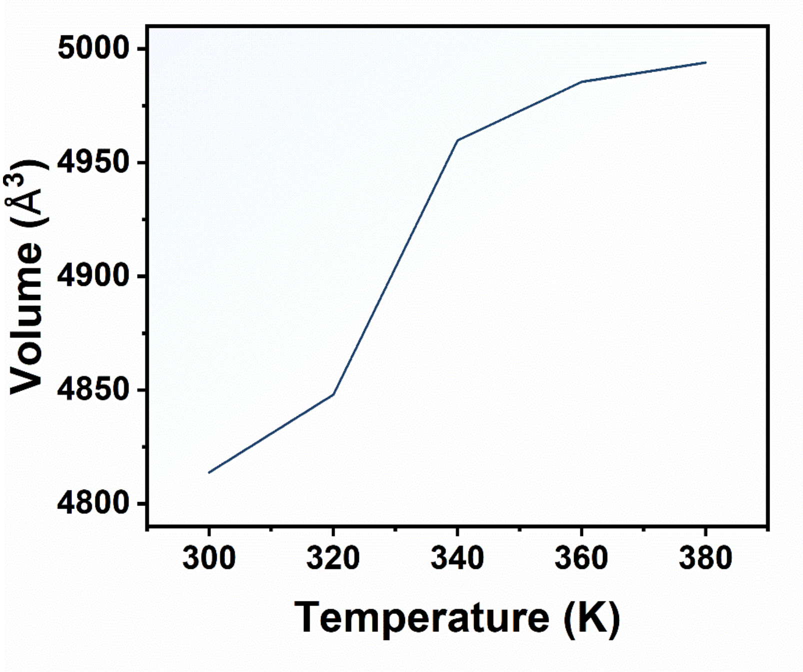

Figure S11. Lattice volume of $TPP_3Cu_2Br_2$ with the temperature from 300 to 380 K. With elevating the temperature from 300 to 380 K, the lattice volume changed from 4818.3 to 4994 Å^3, according to the SCXRD data at different temperatures.

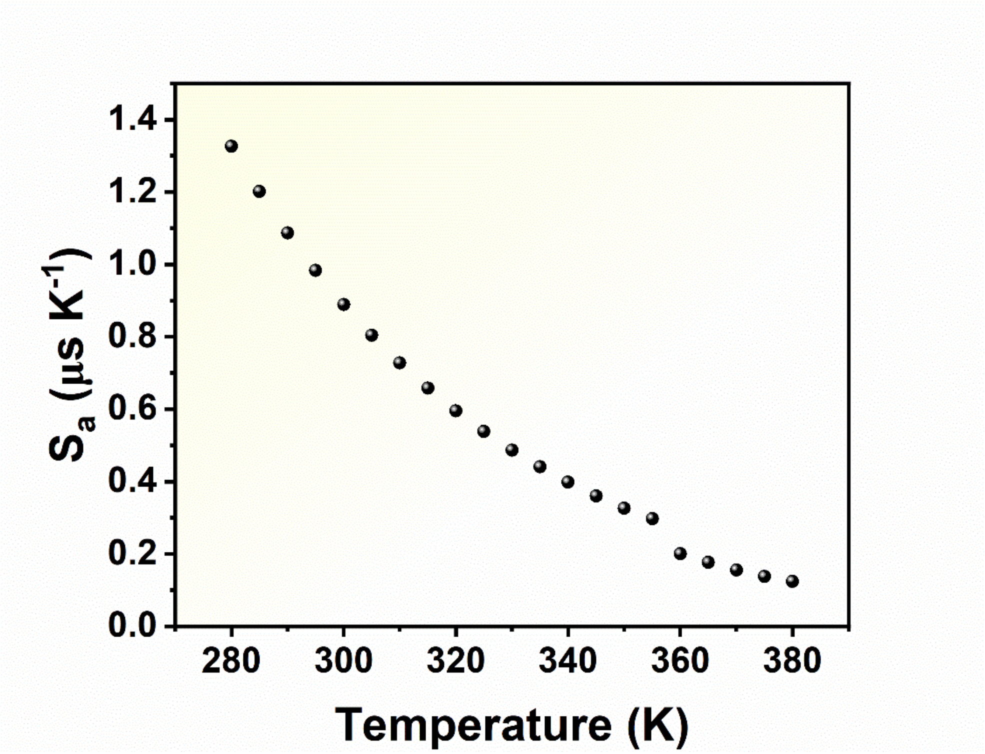

Figure S12. Calculated S_a of $TPP_3Cu_2Br_2$ in the temperature range of 280–380 K based on PL lifetime. The maximum S_a was determined to be 1.33 μs K^{-1} at 280 K.

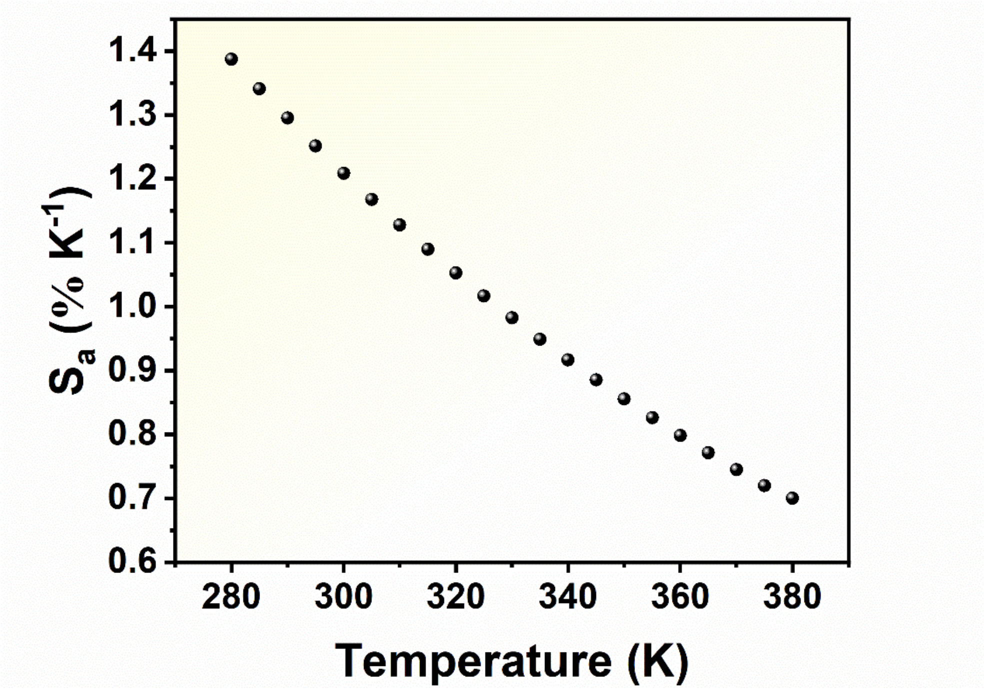

Figure S13. Calculated S_a of $TPP_3Cu_2Br_2$ in the temperature range of 280–380 K based on PL intensity. The maximum S_a was determined to be 1.39 % K^{-1} at 280 K.

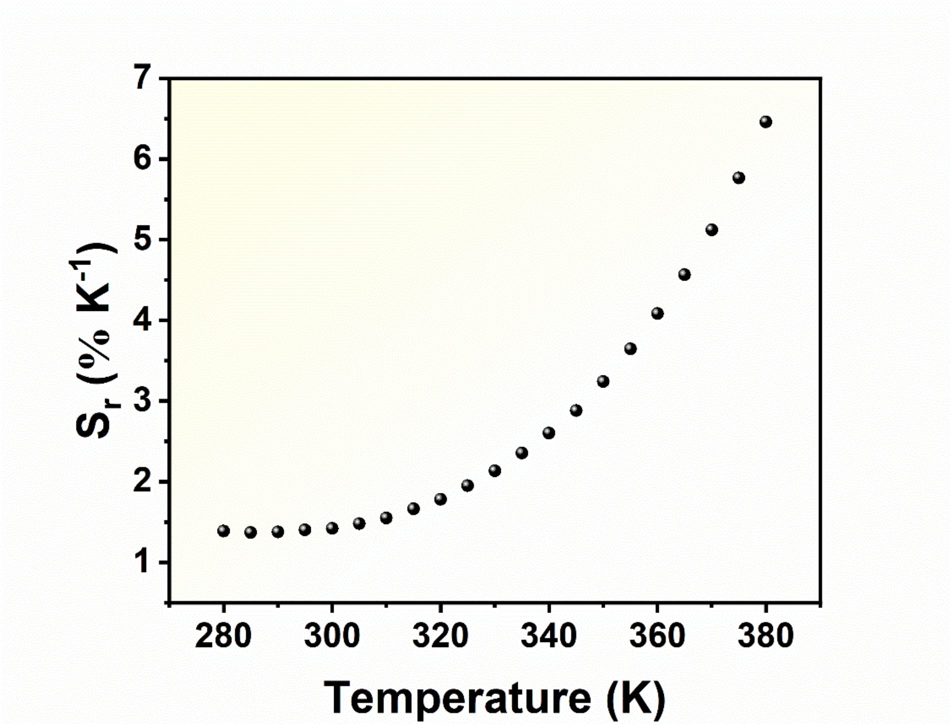

Figure S14. Calculated S_r of $TPP_3Cu_2Br_2$ in the temperature range of 280–380 K based on PL intensity. The maximum S_r was determined to be 6.46 % K^{-1} at 380 K.

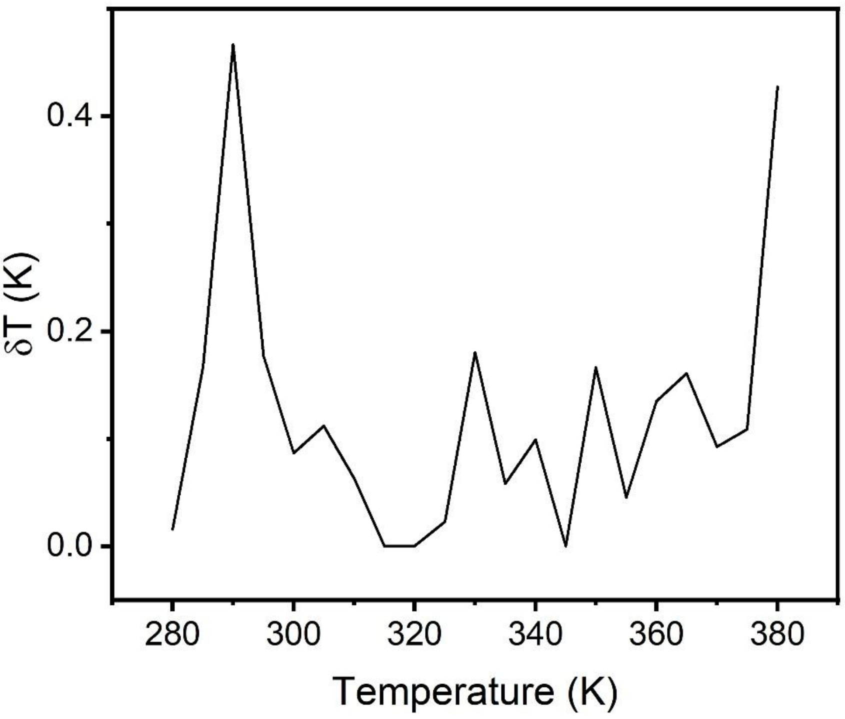

Figure S15. Temperature uncertainty for $TPP_3Cu_2Br_2$ at different temperatures. Accordingly, it was determined that $TPP_3Cu_2Br_2$ exhibited small δT values (≤ 0.47 K) in the range of 280-380 K.

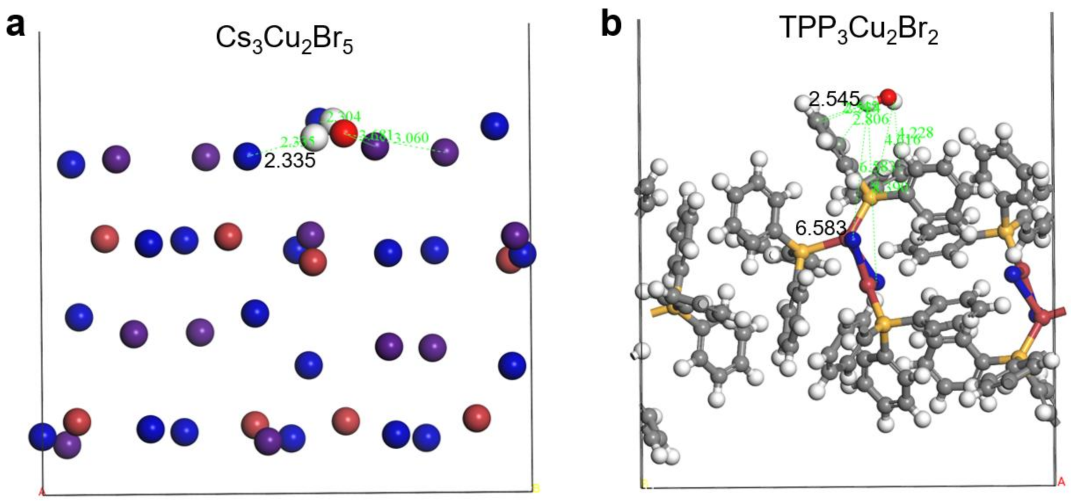

Figure S16. Closest contact distance of a water molecule with a) $Cs_3Cu_2Br_5$ and b) $TPP_3Cu_2Br_2$. The water molecule's closest contact H-Br distance of 2.335 Å with the inorganic $Cs_3Cu_2Br_5$ was smaller than the H-Benzene distance of 2.545 Å with the organic hydrophobic molecule TPP.

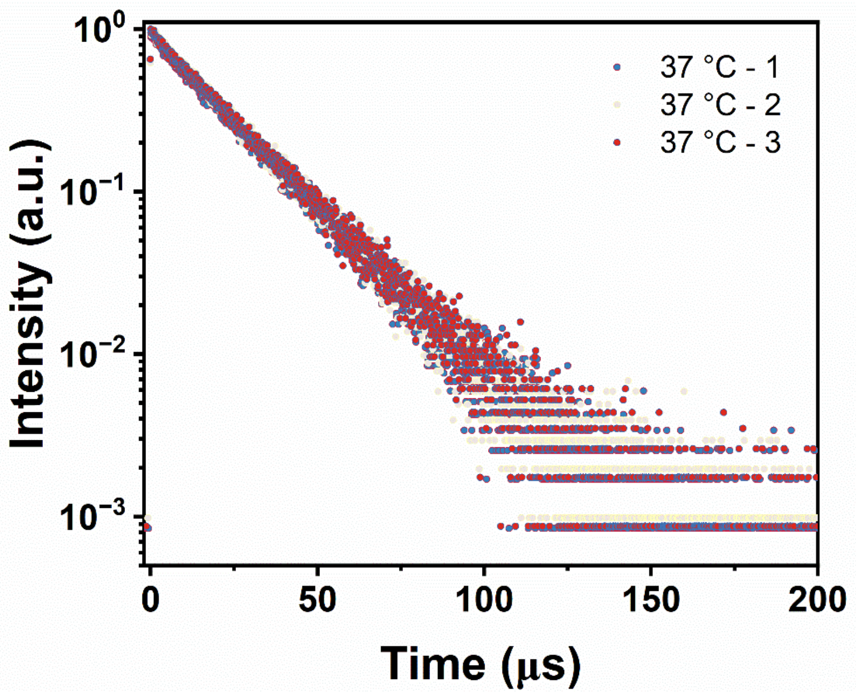

Figure S17. PL decays of the $TPP_3Cu_2Br_2$ in the water of 37 °C by monitoring the emission at 524 nm (λ_{ex} = 355 nm). The average PL lifetime was determined to be 19.83 μs.

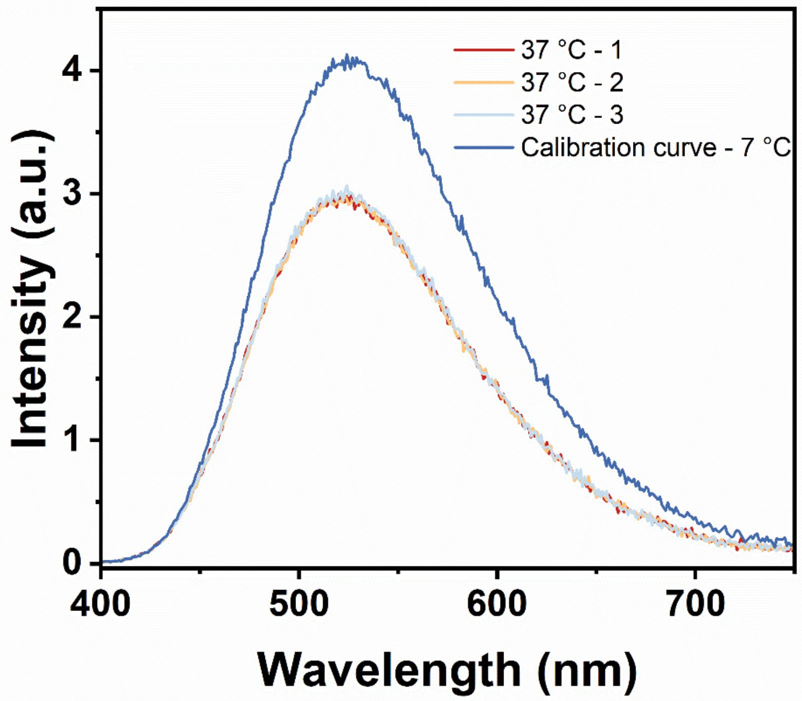

Figure S18. PL emission spectra of the $TPP_3Cu_2Br_2$ in the water of different temperatures upon 355 nm excitation.

Reference

1 Kresse, G. & Furthmüller, J. Efficiency of ab-initio total energy calculations for metals and semiconductors using a plane-wave basis set. *Comput. Mater. Sci.* **6**, 15-50 (1996).
2 Kresse, G. F., J. Efficient Iterative Schemes for Ab Initio Total-Energy Calculations Using a Plane-Wave Basis Set. *Phys. Rev. B* **54**, 11169 (1996).
3 Kresse, G. H., J. Ab Initio Molecular Dynamics for Liquid Metals. *Phys. Rev. B* **47**, 558 (1993).
4 Hafner, J. Ab-Initio Simulations of Materials Using VASP: Density-Functional Theory and Beyond. *J. Comput. Chem.* **29**, 2044 (2008).
5 Kresse, G. J., D. From Ultrasoft Pseudopotentials to the Projector Augmented-Wave Method. *Phys. Rev. B* **59**, 1758 (1999).
6 Blöchl, P. E. Projector Augmented-Wave Method. *Phys. Rev. B* **50**, 17953 (1994).
7 Perdew, J. P. B., K.; Ernzerhof, M. Generalized Gradient Approximation Made Simple [Phys. Rev. Lett. 77, 3865 (1996)]. *Phys. Rev. Lett.* **78**, 1396 (1997).
8 Perdew, J. P. B., K.; Ernzerhof, M. Generalized Gradient Approximation Made Simple. *Phys. Rev. Lett.* **77**, 3865 (1996).
9 Monkhorst, H. J. P., J. D. Special Points for Brillouin-Zone Integrations. *Phys. Rev. B* **13**, 5188 (1976).
10 Filatov, M. & Shaik, S. A spin-restricted ensemble-referenced Kohn–Sham method and its application to diradicaloid situations. *Chem. Phys. Lett.* **304**, 429-437 (1999).
11 Kowalczyk, T., Tsuchimochi, T., Chen, P.-T., Top, L. & Van Voorhis, T. Excitation energies and Stokes shifts from a restricted open-shell Kohn-Sham approach. *J. Chem. Phys.* **138**, 164101 (2013).
12 Frank, I., Hutter, J., Marx, D. & Parrinello, M. Molecular dynamics in low-spin excited states. *J. Chem. Phys.* **108**, 4060-4069 (1998).
13 Hutter, J., Iannuzzi, M., Schiffmann, F. & VandeVondele, J. cp2k: atomistic simulations of condensed matter systems. *Wiley Interdiscip. Rev.: Comput. Mol. Sci.* **4**, 15-25 (2014).
14 Wang, X. *et al.* Atomistic Mechanism of Broadband Emission in Metal Halide Perovskites. *J. Phys. Chem. Lett.* **10**, 501-506 (2019).
15 Luo, J. *et al.* Efficient and stable emission of warm-white light from lead-free halide double perovskites. *Nature* **563**, 541-545 (2018).
16 S. Goedecker, M. T., and J. Hutter. Separable dual-space Gaussian pseudopotentials. *Phys. Rev. B* **54**, 1703 (1996).
17 VandeVondele, J. & Hutter, J. Gaussian basis sets for accurate calculations on molecular systems in gas and condensed phases. *J. Chem. Phys.* **127**, 114105 (2007).
18 Perdew, J. P., Burke, K. & Ernzerhof, M. Generalized Gradient Approximation Made Simple. *Phys. Rev. Lett.* **77**, 3865-3868 (1996).
19 Mayeul d'Avezac, M. C., and Francesco Mauri. Density functional theory description of hole-trapping in SiO_2: A self-interaction-corrected approach. *Phys. Rev. B* **71**, 205210 (2005).
20 Zunger, J. P. P. a. A. Self-interaction correction to density-functional approximations for many-electron systems. *Phys. Rev. B* **23**, 5048 (1981).
21 Morad, V. *et al.* Hybrid 0D Antimony Halides as Air-Stable Luminophores for High-Spatial-Resolution Remote Thermography. *Adv. Mater.* **33**, 2007355 (2021).
22 Li, X. *et al.* Halide Double Perovskite Nanocrystals Doped with Rare-Earth Ions for Multifunctional Applications. *Adv. Sci.* **10**, 2207571 (2023).
23 Wei, J. H. *et al.* Te^{4+}-doped $Cs_2InCl_5·H_2O$ single crystals for remote optical thermometry. *Sci. China Mater.* **65**, 764-772 (2021).
24 Wu, L.-K. *et al.* Te^{4+}-Doping Rubidium Scandium Halide Perovskite Single Crystals Enabling Optical Thermometry. *J. Phys. Chem. C* **126**, 21689-21698 (2022).
25 Li, G. *et al.* Regulating Exciton De-Trapping of Te^{4+}-Doped Zero-Dimensional Scandium-Halide Perovskite for Fluorescence Thermometry with Record High Time-Resolved Thermal Sensitivity. *Adv. Mater.* **35**, 2305495 (2023).
26 Zhao, S. *et al.* Luminescent enhancement and multi-mode optical thermometry of erbium doped halide $Cs_2(Na/Ag)BiCl_6$ microcrystals. *J. Rare Earths* **42**, 2018-2026 (2024).
27 Benin, B. M. *et al.* The $Rb_7Bi_{3-3x}Sb_{3x}Cl_{16}$ Family: A Fully Inorganic Solid Solution with Room-Temperature Luminescent Members. *Angew. Chem. Int . Ed.* **59**, 14490-14497 (2020).
28 Bai, Y., Zhang, S., Luo, N., Zou, B. & Zeng, R. Temperature-dependent self-trapped exciton emission in Cu(I) doped zinc-based metal halides from well-resolved excited state structures. *Nano Res.* **17**, 7768-7775 (2024).
